# Perspectives on microbial community changes produced by *Hermitia illucens* frass and their impact on soil suppression against *Fusarium oxysporum* f. *sp. lactucae*

**DOI:** 10.1002/ps.70036

**Published:** 2025-07-08

**Authors:** Paloma Hernández‐Muñiz, Celia Borrero, Manuel Avilés, Jesús Dionisio Fernández‐Bayo

**Affiliations:** ^1^ Departamento de Agronomía, Escuela Técnica Superior de Ingeniería Agronómica Universidad de Sevilla Seville Spain; ^2^ Department of Biological and Agricultural Engineering University of California Davis Davis California USA; ^3^ Departamento de Edafología y Química Agrícola Universidad de Granada Granada Spain

**Keywords:** insect frass, chitin, disease suppression, black soldier fly larvae, biocontrol agents, fusarium wilt

## Abstract

**BACKGROUND:**

Organic soil amendments rich in chitin have demonstrated significant potential promoting suppressive soils. Suppressive soils inhibit the growth and activity of soilborne pathogens, being a sustainable alternative to chemical fumigation. The aim of this study was to determine the ability of frass produced from black soldier fly larva (BSFL), a novel chitin‐rich soil amendment, and other amendments enriched in chitin to promote suppressive soils against *Fusarium oxysporum* f. *sp. lactucae* (*Fol*) and to assess the role of the microbial community on suppressiveness.

BSFL frass, compost, chitin and a mixture of compost and chitin were mixed with soil. Some soil mixes were incubated for 4 months to promote further changes in the microbial community structure. Then, incubated and non‐incubated soil mixes were inoculated with *Fol* to study the reduction of disease severity caused by *Fol* on lettuce and the evolution of soil microbial communities.

**RESULTS:**

Soils that were incubated with chitin or BSFL frass showed a significant decrease in *Fol* population density and disease severity compared to the unamended and non‐incubated soils (*P* < 0.05). Analyses of microbial community of soils incubated with chitin and BSFL frass were compared to the non‐amended soil, they were enriched on fungal and bacterial OTUs of microorganisms known as biocontrol agents such as *Mortierellales*, *Trichoderma spp.*, *Chaetomium globosum, Streptomyces*, *Bacillus* and *Sphingomonas*.

**CONCLUSION:**

Soil incubated with chitin and BSFL frass seem to promote suppressiveness against *Fol*. Further studies on chitin or BSFL frass concentration, incubation period or amendment rate are needed to improve soil suppressiveness. © 2025 The Author(s). *Pest Management Science* published by John Wiley & Sons Ltd on behalf of Society of Chemical Industry.

## Graphical abstract

Soils amended with chitin or black soldier fly larvae (BSFL) frass reduced *Fusarium oxysporum f. sp. lactucae* propagules compared to non‐amended soil. Changes observed in microbial communities could be involved.

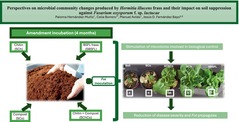

## 1 INTRODUCTION

According to the Food and Agriculture Organization of the United Nations (FAO), it is estimated that between 20% and 40% of the world's crop production is lost annually due to pests such as soil pathogens. Among those causing the greatest economic losses in horticultural crops is *Fusarium oxysporum*. Specifically, in lettuce cultivation it is attacked by *Fusarium oxysporum* f. *sp. lactucae* (*Fol*) and causes Fusarium wilt.^1^ This pathogen affects lettuce production worldwide and once established in a field, is difficult to eradicate, as the cost of chemical fumigation is economically prohibitive for lettuce producers.^2^ In addition, chemical soil fumigation poses significant environmental and human health problems and, consequently, has forced regulators to be stricter and to ban efficient fumigants such as methyl bromide.^3^ This raises the urgent need to find less harmful sustainable alternatives to chemical fumigation.

The development of suppressive soils is considered as a significant sustainable alternative to chemical fumigation. The inhibition of the growth and activity of soil pathogens by suppressive soils is attributed to two main mechanisms, the specific activity of the microorganisms present in the soil or substrates, and their competitive and antagonistic activity against pathogens.^4^, ^5^, ^6^ Several factors such as pH and the β‐glucosidase activity of microbial populations, are considered predictive variables for the suppression of Fusarium wilt disease.^7^

A common practice among farmers to change soil composition and induce the growth and activity of some of the microorganisms that promote soil suppression is the addition of specific organic amendments.^8^ In particular, chitin‐rich organic soil amendments have shown significant potential in promoting suppressive soils.^9^ Chitin amendments have biopesticidal properties based on the induction of systemic resistance in plants,^10^ thus improving their natural mechanism against pathogens.^11^ Chitin amendment can also stimulate the growth of chitinolytic microorganisms in the soil after the addition of the chitin amendment.^12^

During the digestion of agro‐industrial organic by‐products using black soldier fly larvae (BSFL), a promising suppressive soil amendment is produced due to the presence of chitin during the process. BSFL larvae contain chitin in the exoskeletons that they shed in the substrate after molting.^13^ This soil amendment, known as frass, is composed of larval excrement, undigested material and chitin. Research has shown that BSFL frass has antifungal properties against plant pathogens such as *Fusarium sp*.^14^, ^15^, ^16^ and *Ralstonia solanacearum* (Smith).^17^ In addition, this amendment can also increase the yield of some crops when applied to potting soil.^18^, ^19^, ^20^ Therefore, it is interesting to continue investigating the potential of BSFL frass as a possible suppressive soil promoter.

The aim of this study was to determine the capacity of BSFL frass, in contrast to other soil amendments also enriched in chitin, to promote suppressive soils against lettuce Fusarium wilt. In addition, the microbial communities evolved in the soil amended with these amendments were characterized to identify the possible microorganisms responsible for this suppression.

## 2 MATERIALS AND METHODS

### 2.1 Experimental design

Three trials were developed to evaluate and study the capacity of different amendments to promote soil suppressiveness against lettuce Fusarium wilt (trials 1 and 2) and to assess phytotoxicity (trial 3) of amendments in lettuce plants. Each trial was conducted in triplicate.

Trials consisted of five treatments with three repetitions and were carried out in a greenhouse. Trials 1 and 2 were artificially inoculated with *Fol*. Trial 1 was incubated with amendments for 4 months before the experiment to modify the initial soil community structure, and trials 2 and 3 were not incubated.

### 2.2 Preparation of the substrates for lettuce growth experiments

The soil sample was collected from the research farm of the Department of Plant Pathology at the University of California, Davis (USA). The field soil texture was determined to be 47% sand, 27% silt, and 26% clay. The organic matter content and the field capacity were 2.64% and 21.90%, respectively (wet basis).

The amendments used and mixed with the soil were: commercial chitin from crab meal (SCh) at a rate of 0.2% (dry weight basis)^21^ (Down to Earth Organic Crab Meal Mix 4–3‐0, Eugene, Oregon, USA); compost (SCo) (R&M Organics Premium Compost 1.25‐1‐2.5, Wasco, California, USA) and BSFL frass (SBSFL) (BioMilitus LLC, Davis, USA). BSFL were added at a rate of 2% (d.w.).^22^ The initial diet of the larva included a mix of almond hulls and shells, brewed grain and hydrolyzed food waste.

To evaluate short‐ and long‐term changes in microbial communities, a set of soils (amended and unamended) was prepared 4 months before the experiment (incubated trial 1) and one week before the start of the experiment (unincubated trials 2 and 3). Both trials were incubated in buckets in a greenhouse at 18–32°C temperature and at a water content of 60% of field capacity. Soils from the 4‐month incubated trial were maintained at water content by correcting mass loss with water every 2 weeks. To facilitate proper mixing with the inoculum, 2 weeks prior to inoculation with *Fol*, incubated soils were allowed to dry and mixed every 2 days to break up aggregates. In trial 2 the soils were amended and inoculated with *Fol* at the same time. In trial 3, only the amendment (without pathogen) was added. Trial 3 was used to check for any possible phytotoxic effects of the newly added amendments.

Prior to the beginning of the trials, the amended and unamended soils of each treatment were combined with perlite in a 1:1 ratio and distributed into containers for each trial.

### 2.3 Suppressiveness and phytotoxicity bioassays

The inoculum was prepared with a strain of *Fol* race 1. The isolate was provided by the Department of Plant Pathology at the University of California, Davis (USA) and grown in Potato Dextrose Agar (PDA) medium for one week at room temperature. Conidia inoculum suspension was prepared according to Borrero *et al*.^7^

Perlite‐mixed soils (amended and unamended) from each container of trials 1 and 2 were artificially inoculated at a concentration of 4.53 × 10^5^ conidia/g dry soil of *Fol*. Then, soils were mixed and watered to 60% of their field capacity. They were left for 2 days in the greenhouse to allow proper establishment of the pathogen. *Fol* was established at a similar concentration in both trials, 3.45 × 10^5^ conidia/g dry soil (Section 2.4).

After pathogen establishment, three 0.5‐L plant pots were filled from each container of each treatment. One 25 days‐old lettuce plant grown in a seedbed was planted per pot. The Buttercrunch (*Lactuca sativa*) lettuce cultivar was selected for the experiment because of its sensitivity to *Fol* race 1 and its tolerance to high temperatures. Prior to the trials, lettuce plants were grown in the greenhouse for 25 days at a temperature range of 18–32 °C. Plants were fertilized twice a week.

Disease severity was measured using a Fusarium wilt scoring scale ranging from 0 to 5 [0 = no disease, 1 = slight stunting and chlorosis, 2 = moderate stunting and chlorosis, 3 = wilting and chlorosis, 4 = severe wilting and chlorosis, 5 = dead plant].^23^ The discrete scale score was converted to the midpoint of the corresponding disease severity range prior to using parametric analyses. The area under the disease progress curve standardized (AUDPC) per experimental unit was calculated by integrating the disease severity values by the trapezoidal integration method between onset of symptom and bioassay end time and dividing by the total epidemic duration (days) in each bioassay.^24^ Therefore, AUDPCs data ranged from 0 to 1.

Lettuce biomass was weighted in all trials. The biomass of lettuce in trial 3 was used as an indicator of phytotoxicity of the amendments. At the end of the trial, the lettuce from each pot was harvested and the fresh biomass was recorded. Dry biomass was recorded after drying the material at 60 °C until constant weight.

### 2.4 Characterization of lettuce growth soils

Before the start of the suppressiveness trials (Trial 1 and 2) measurements of pH and electrical conductivity (EC) were determined using a pH‐meter (SevenCompact S220, Mettler Toledo, OH, USA) and an electrical conductivity meter (SevenCompact S230, Mettler Toledo, OH, USA), respectively. For that, 7 g of air‐dried soil were taken from each container of each treatment of each trial, mixed with 7 mL of distilled water (1:1, mass of sample d.w./mass water) in 15‐mL tubes and shaken for 30 min.

The quantification of *Fol* population densities as CFU/ g dry soil was performed before and after the suppressiveness trials. Prior to the beginning of the trials, a sample was taken from each container, and, after plantation, one rhizosphere soil (adhered soil to roots after one shake) sample was taken from each repetition (three samples per treatment). The methodology followed was: air‐dried soil samples (10 g) were suspended in 200 mL of 1% sodium hexametaphosphate and stirred for 5 min. Ten mL of the suspension were then diluted in 0.1% water agar (1:10) and agitated for another 5 min.^25^ Three serial dilutions were carried out and, finally, 400 μL of different dilutions were spread in triplicate to Komada's selective medium plates.^26^ Plates were incubated at room temperature under continuous fluorescent light for 10–14 days. Colonies were identified based on morphology as described by Scott *et al*.^27^

### 2.5 DNA extraction and 16S rRNA and ITS gene sequencing

Subsamples of incubated (trial 1) and non‐incubated (trial 2) soils were taken from each container before mixing the substrate with the perlite (before the start of the plant trial) and stored at −20 °C. Genomic DNA was extracted and purified from each subsample (one replicate per treatment and trial) using a commercial DNA Isolation Kit (DNeasy PowerSoil Kit, Qiagen, Hilden, Germany). Sequencing of the extracted bacterial DNA was done using MiSeq amplicon sequencing (Illumina, San Diego, CA) by RTL Genomics (Lubbock, TX) according to their pipeline. For bacterial analyses, sequences corresponding to the V4‐V5 hypervariable region of 16S rRNA using the primer pairs 515yF GTGYCAGCMGCCGCGGTAA and 926pfRCCGYCAATTYMTTTRAGTTT was used. The sequences used for fungal analyses were ITS1F CTTGGTCATTTAGAGGAAGTAA, and ITS2 aR GCTGCGTTCTTCATCGATGC. The RTL genomic pipeline used high‐quality sequences derived from NCBI to make the taxonomic classification. Singleton OTUs with one read were removed prior to analysis.

### 2.6 Data analysis

The incubation and non‐incubation trials were analyzed separately, given that in a preliminary analysis the interaction between incubation × treatment was significant for both dependent variables (severity of Fusarium wilt and population densities of *Fusarium oxysporum* f. *sp. lactucae* after lettuce crop). The trials were repeated three times. The experimental design was completely randomized with three replicates. The experimental unit was a pot with a lettuce plant. The dependent variables were analyzed by ANOVA. The factors included were treatment, trial and replicate nested in trial and their interactions. As the treatment × trial interactions were not significant, the results include the data set of the three trials. For the variables pH and electrical conductivity, only one measurement per treatment and trial was taken from one aliquot of the three replicates included in each trial. In these cases, the analysis was an ANOVA with the factor's treatment and trial. The separation of means for the factors that were significant was performed by LSD test (*P* < 0.05). The requirements for ANOVA were checked by Levene's test and, when necessary, data were transformed. All data analyses were performed using Statgraphics Centurion 18 (version 18.1.13; Statgraphics Technologies, Inc., The Plains, VA) and JMP (version 16.1.0, SAS Campus Drive, Cary, NC, USA).

RStudio Version 2022.12.0 + 353 (Posit Software, PBC) was used to determine microbial taxonomy relative abundance, operational taxonomic units (OTU), pielou evenness, and Shannon diversity index. The Vegan package version 2.6‐4^28^ was used to generate the nonmetric multidimensional scaling (NMDS) plots from the Bray–Curtis dissimilarity matrix (calculated from OTU abundance values) based on 1000 random starts. The Adonis function in vegan was used to perform two‐way permutational multivariate ANOVA (PERMANOVA) using the Bray–Curtis dissimilarity matrix with 999 permutations to determine soil treatment and incubation effects in the NMDS plots. Similarity percentage (SIMPER) analysis was carried out to identify specific OTUs that contributed most to observed Bray–Curtis dissimilarity.^29^

## 3 RESULTS

### 3.1 Impact of the amendments on the soil pH and electrical conductivity

In the incubated soils (trial 1), SChCo and SBSFL showed no significant differences between them, and recorded the highest electrical conductivity (EC) values (*P < 0.05*, Table 1). In contrast, NAS had the lowest EC and was significantly different from the other treatments. SCo and SCh showed intermediate EC values, however, they also differed significantly from each other and from the other treatments (*P < 0.05*, Table 1).

**Table 1:** Electrical conductivity (EC) and pH measured in the soils incubated for 4 months (trial 1) and not incubated (trial 2) before lettuce crop

^†^Treatments	Trial 1 (incubated soil)		Trial 2 (non‐incubated soil)	
	EC (μS/cm)	pH	EC (μS/cm)	pH
NAS	409.67 ± 3.85 d	6.80 ± 0.02 b	604.67 ± 22.40 c	6.64 ± 0.00 cd
SCo	986.33 ± 18.56 b	6.84 ± 0.02 b	1175.67 ± 73.45 a	6.93 ± 0.01 a
SCh	739.0 ± 11.14 c	6.85 ± 0.01 b	906.33 ± 47.24 b	6.65 ± 0.07 bc
SCoCh	1213.00 ± 38.18 a	6.93 ± 0.01 a	1212.67 ± 13.45 a	6.82 ± 0.01 b
SBSFL	1148.67 ± 33.20 a	6.28 ± 0.01 c	667.67 ± 13.91 c	6.60 ± 0.02 d

^**†**^ Treatments: NAS, non amended soil; SCo, soil + compost; SCh, soil + chitin; SCoCh, soil+ compost+ chitin; SBSFL, soil + Black soldier fly larva (BSFL) frass. Data of EC were transformed as x^−1^ for trial 1 and x^0.3^ for trial 2.

Data represents the mean ± standard error (*n* = 3). Values followed by different letters indicate significant differences (*P* < 0.05) according to LSD test for all parameters.

In the non‐incubated soils (trial 2), SCo and SCoCh showed significantly highest EC values (*P < 0.05*), followed by SCh with intermediate EC values and the lowest EC were observed in NAS and SBSFL (*P < 0.05*). The pH was significantly highest in SCo and continued being significantly lowest in SBSFL (*P < 0.05*, Table 1).

### 3.2 Impact of short‐term and long‐term chitin‐rich amendments addition to soil on *Fol* suppression

**Figure 1:**
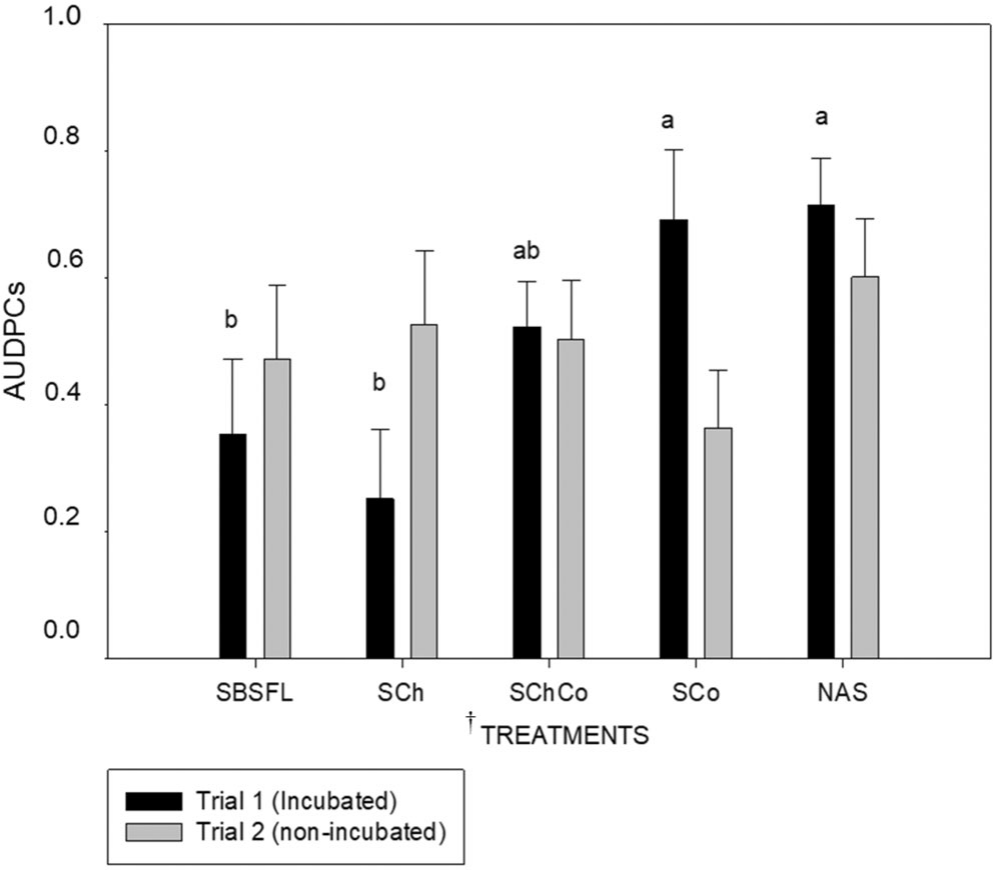
Standardized area under disease progress curve (AUDPCs) for lettuce Fusarium wilt severity in Trial 1 (incubated soils; black bars) and Trial 2 (non‐incubated soils; grey bars). ^†^Treatments: SBSFL, Soil+ Black soldier fly larva (BSFL) frass; SCh, soil + chitin; SCoCh, soil+ compost+ chitin; SCo, soil + compost; NAS, non amended soil. Bars with different letters indicate significant differences within treatments for the same incubation duration according to LSD test at *P < 0.05*. Standard error of mean is indicated by vertical line (*n* = 9).

The AUDPCs in lettuce plants was significantly affected by the amendment type in the incubated soils (trial 1) (*P < 0.05*, Fig. 1). In Trial 1, it was observed that the soils with the lowest AUDPC values, and therefore considered the most suppressive against *Fol*, were those amended with BSFL (SBSFL) and chitin (SCh). These soils showed significant differences compared to the soil amended with compost (SCo) and the non‐amended soil (NAS). Additionally, all these soils were statistically similar to the soil amended with the chitin and compost mix (SChCo) (*P < 0.05*, Fig. 1).

In trial 2, no statistical differences between amendments were observed (Fig. 1).

**Figure 2:**
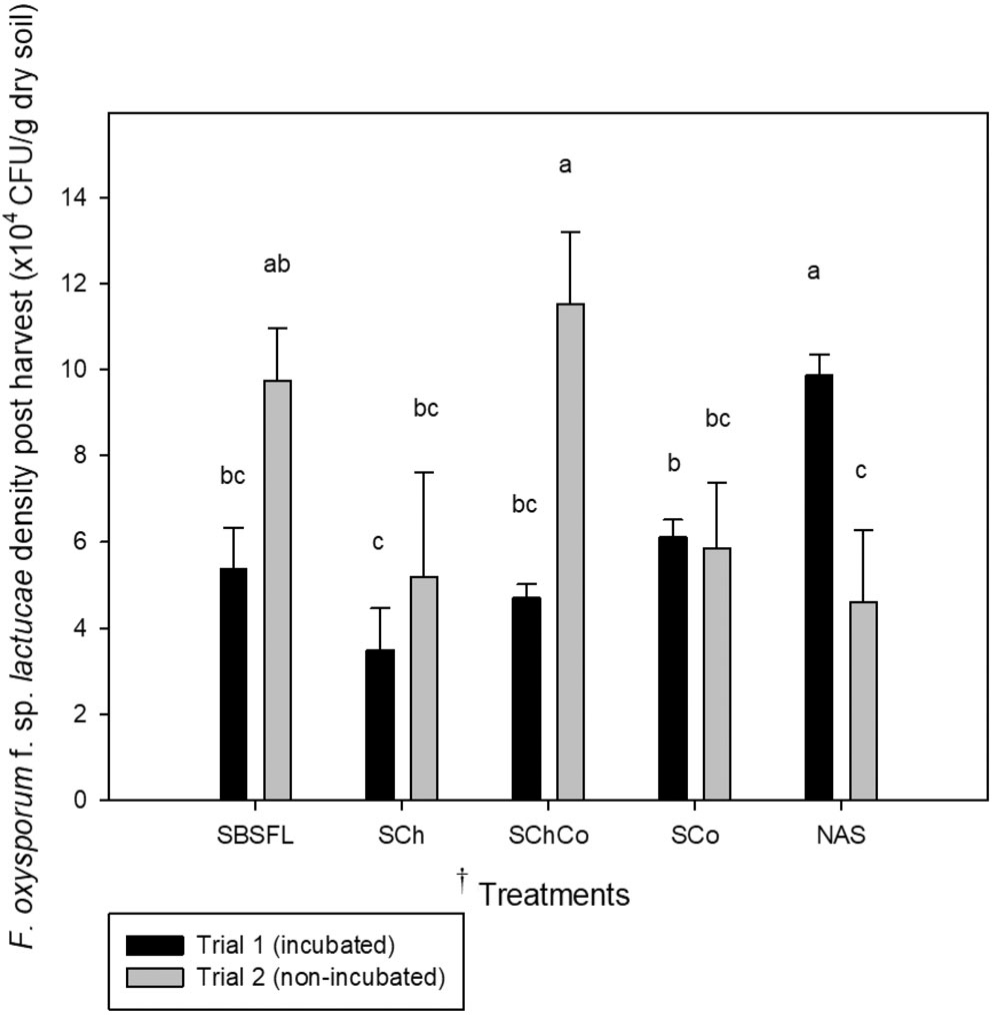
*Fusarium oxysporum* f. *sp. lactucae* population densities after lettuce crop in Trial 1 (incubated soils) and Trial 2 (non‐incubated soils). ^†^Treatments: SBSFL: Soil+ Black soldier fly larva (BSFL) frass; SCh: soil + chitin; SCoCh: soil+ compost+ chitin; SCo: soil + compost; NAS: non amended soil. Bars with different letters indicate significant differences within treatments for the same incubation duration according to LSD test at *P* < 0.05. Standard error of mean is indicated by vertical line (*n* = 9).

After the experiments with lettuce, in the incubated treatments (Trial 1), a significant decrease in *Fol* population density was observed in all amended treatments compared to the non‐amended soil (NAS, *P < 0.05*). The SCh treatment exhibited the lowest value of *Fol* populations, however, the value was not significantly lower than SBSFL and SChCo. The SCo treatment showed intermediate *Fol* populations levels (Fig. 2).

In the non‐incubated trial (Trial 2), no reduction in *Fol* population density was observed in the amended soils when compared to the non‐amended soil (NAS). SCh and SCo showed statistically the same level of CFU/g dry soil as the control (NAS). In addition, these treatments did not show significant differences with SBSFL. In turn, SChCo and SBSFL recorded the highest CFU values that were significantly higher than the NAS (Fig. 2).

### 3.3 Lettuce biomass

Dry lettuce biomass per plant was significantly affected by the amendment type in the incubated soil (Trial 1) (*P* < 0.05, Table 2). The incubated soil amended with chitin (SCh) showed significantly higher biomass than all the treatments except SBSFL (*P* < 0.05). Lettuce grown on SBSFL showed the second highest biomass mean value that was significantly higher than the control NAS (*P < 0.05*). NAS, SCo and SChCo did not show significant difference on lettuce biomass (Table 2).

**Table 2:** Dry weight of the lettuce aerial biomass grown on trials 1, 2 and 3

^†^Treatments	^‡^Trial 1 dry weight (g)	^§^ Trial 2 dry weight (g)	^¶^ Trial 3 dry weight (g)
NAS	0.76 ± 0.29 c	1.01 ± 0.34	4.57 ± 0.32
SCo	1.21 ± 0.43 bc	2.34 ± 0.47	4.58 ± 0.27
SCh	3.00 ± 0.56 a	1.37 ± 0.53	4.11 ± 0.33
SBSFL	2.41 ± 0.60 ab	1.73 ± 0.49	4.86 ± 0.27
SChCo	1.60 ± 0.33 bc	1.49 ± 0.47	4.07 ± 0.27

^**†**^ Treatments: NAS, non‐amended soil; SCo, soil + compost; SCh, soil + chitin; SBSFL, Black soldier fly larva (BSFL) frass; SCoCh: soil + compost + chitin.

^‡^ Trial 1: incubated and inoculated trial;

^§^ Trial 2. non‐incubated and inoculated trial.

^¶^ Trial 3: non‐incubate and non‐inoculated trial.

Data represents the mean ± standard error. Values followed by different letters in each column indicate significant differences according to ANOVA and LSD test (*P* < 0.05), *n* = 9 for dry weight.

Lettuce grown in soils that were freshly amended without *Fol* inoculum to test for any direct phytotoxic effect (Trial 3) from the amendments, also did not show statistically significant difference among the treatments (Table 2). This indicates that no direct phytotoxic effect of the amendments used was expected to affect the results on the lettuce biomass results on soils inoculated with *Fol*. The lettuce biomass was statistically similar within treatments in the non‐incubated soil (Trials 2 and 3; Table 2).

### 3.4 Characterization of the soil fungal community

#### 3.4.1 Diversity and structure of the fungal community

Both, Pielou and Shannons diversity index of the fungal community were not affected neither by the treatment nor the incubation of the soils (*P > 0.05*, Table 3).

**Table 3:** Mean and standard deviation of the fungal and bacterial Shannon and Pielou diversity index

Incubation		Fungi		Bacteria	
	^†^Treatments	Shannon	Pielou	Shannon	Pielou
^‡^Trial 1 (Incubated)	NAS	1.48 ± 0.07	0.30 ± 0.02	4.70 ± 0.17bc	0.68 ± 0.03bcd
	SCo	2.00 ± 0.19	0.40 ± 0.04	4.82 ± 0.27bc	0.69 ± 0.01bcd
	SCh	1.97 ± 0.26	0.41 ± 0.06	4.72 ± 0.07bc	0.65 ± 0.01d
	SChCo	2.48 ± 0.40	0.49 ± 0.07	4.87 ± 0.12bc	0.68 ± 0.01bcd
	SBSFL	1.59 ± 0.64	0.32 ± 0.13	5.01 ± 0.06ab	0.71 ± 0.01bc
^§^Trial 2 (Non‐incubated)	NAS	1.60 ± 0.37	0.31 ± 0.09	4.88 ± 0.07bc	0.70 ± 0.01bc
	SCo	1.80 ± 0.47	0.34 ± 0.10	5.25 ± 0.05a	0.75 ± 0.01a
	SCh	1.73 ± 0.12	0.32 ± 0.02	4.54 ± 0.05c	0.65 ± 0.01d
	SChCo	1.80 ± 0.26	0.34 ± 0.05	4.93 ± 0.09ab	0.71 ± 0.01ab
	SBSFL	1.76 ± 0.62	0.34 ± 0.11	4.81 ± 0.12bc	0.67 ± 0.02 cd

^**†**^ Treatments: NAS, non‐amended soil; SCo, soil + compost; SCh, soil + chitin; SBSFL, Black soldier fly larva (BSFL) frass; SCoCh: soil+ compost+ chitin.

^‡^ Trial 1: incubated and inoculated trial;

^§^ Trial 2: non‐incubated and inoculated trial.

Different letters in the same column indicate significant differences within samples (*P* < 0.05), absence of letters indicates no significant differences.

**Figure 3:**
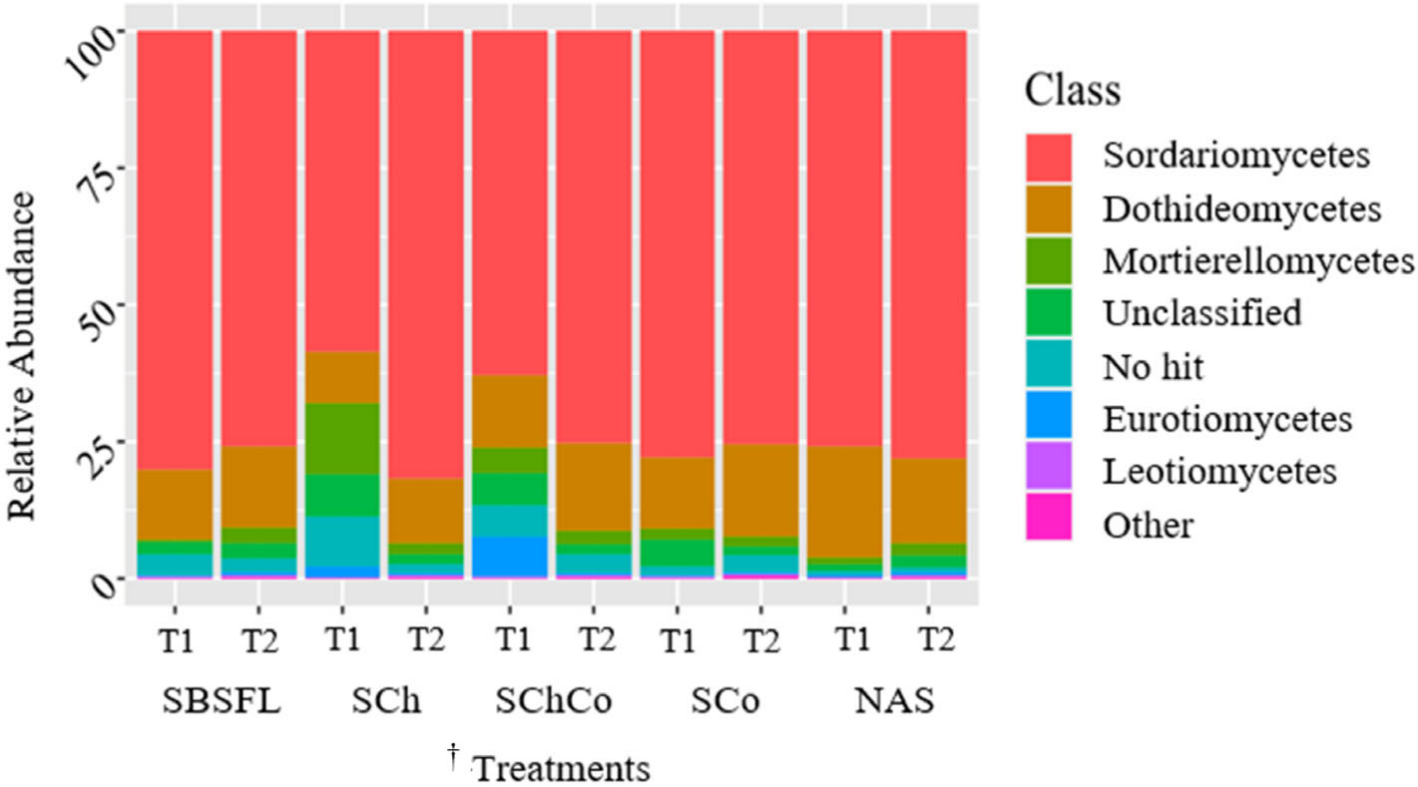
Relative abundance (%) of the main fungal classes measured in the incubated (Trial 1) and non‐incubated soil (Trial 2). ^†^Treatments: SBSFL, Soil+ Black soldier fly larva (BSFL) frass; SCh, soil + chitin; SCoCh, soil+ compost+ chitin; SCo, soil + compost; NAS, non amended soil non‐amended (NAS) or amended with frass from black soldier fly larvae (SBSFL), chitin (SCh), compost (SCo) or compost and chitin (SChCo).

At the phylum level only four phyla were identified (Ascomycota, Mucoromycota, Basidiomycota, and Olpidiomycota), integrating on average 94.3% of the fungal diversity in all samples. Therefore, for fungal diversity, the taxonomy was analyzed at the class level. The most abundant classes were Sordariomycetes, Dothideomycetes, Mortierellomycetes, Eurotiomycetes, and Leotiomycetes, (Fig. 3, Table S1). Mortierellomycetes and Eurotiomycetes were significantly affected by the incubation time, the amendment type and their interaction (*P < 0.05*, Table S1). Particularly, Mortierellomycetes relative abundance was significantly highest in the soil incubated with chitin amendment (SCh, *P < 0.05*, Table S1). Incubation of samples with chitin alone or chitin and compost significantly increased the relative abundance of Mortierellomycetes and Eurotiomycetes (*P < 0.05*, Table S1).

**Figure 4:**
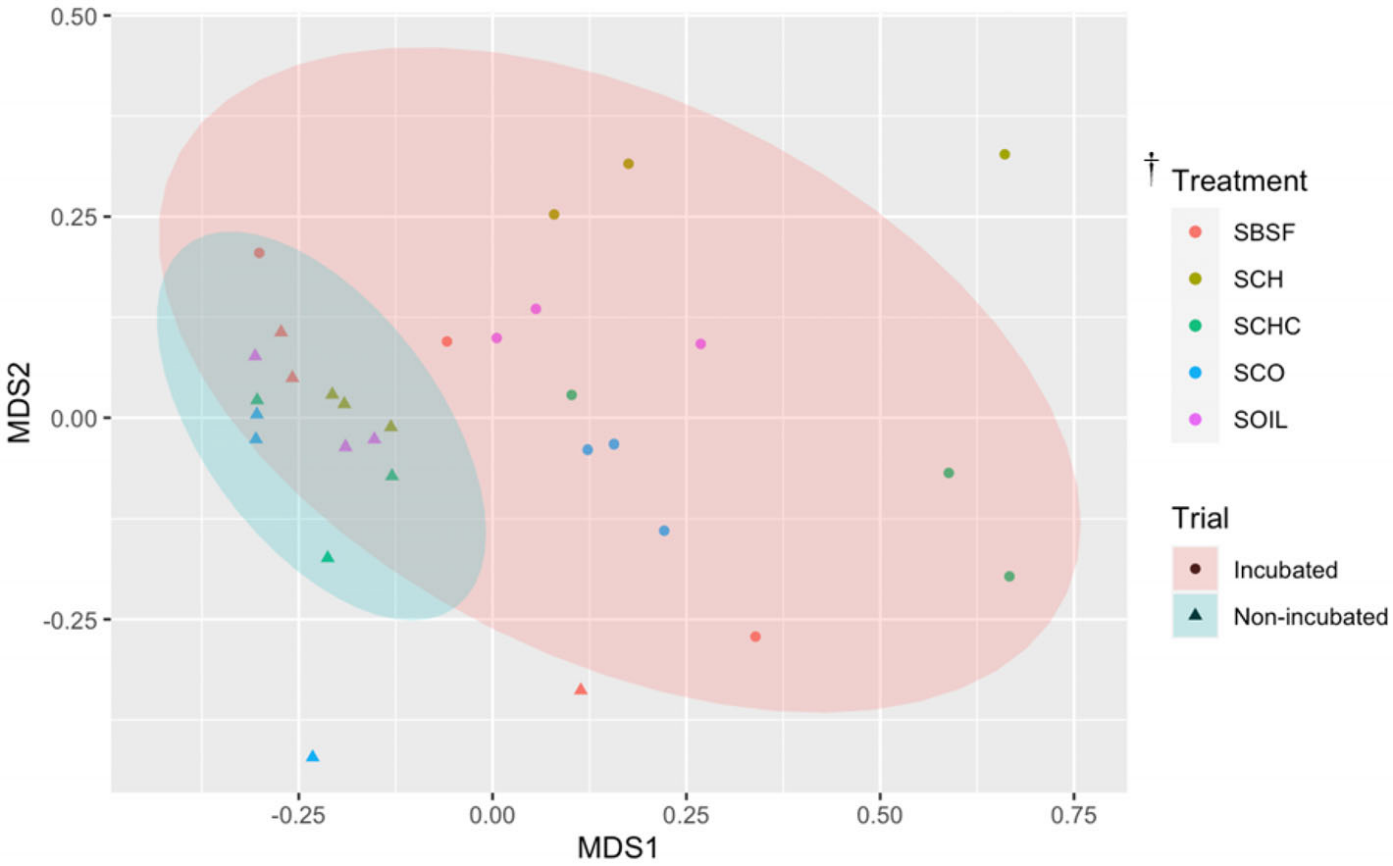
Non‐metric Multidimensional Scaling (NMDS) of the Bray Curtis dissimilarity of the fungal community in the non‐incubated and incubated soil. ^†^Treatment: SOIL, non‐amended soil; SCo, soil + compost; SCh, soil + chitin; SBSFL, Soil + Black soldier fly larva (BSFL) frass; SCoCh, soil+ compost+ chitin. Ellipses represent 95% confidence intervals for the centroids associated with each cluster related to the samples that were non‐incubated or incubated for 4 months.

Finally, the Non‐metric Multidimensional Scaling (NMDS) plot of the fungal community did not show sharps differences within treatments (Fig. 4). Some differentiation was observed between samples that were incubated.

#### 3.4.2 Similarity percentages (Simper) of the main OTUs of the fungal community in the incubated non‐amended soil compared to the soils amended with chitin or BSFL frass

Simper analyses of the fungal communities were only done comparing the incubated non‐amended soil against the incubated soils amended with chitin or BSFL frass because they showed some control effect on *Fol* (Figs 1 and 2).

The fungal OTUs with the highest abundance in chitin‐amended soil (SCh) compared to unamended soil (NAS) belonged to the order *Mortierellales* (OTU 22), *Aspergillus versicolor* (OTU 416), *Penicillium lanosum* (OTU 162), species of the genera *Fusarium* (OTU 185, 336, 477) and *Trichoderma atroviride* (OTU 120). A decrease in the relative abundance of *Fusarium oxysporum* was also observed (Table 4).

**Table 4:** Similarity Percentages (Simper) of the 20 top fungal OTUs of the incubated soil (trial 1) non‐amended compared to the soils amended with chitin (SCh) or black soldier fly larvae frass (SBSFL)

OTU	Species	Relative abundance (%)		^†^CumSum (%)
		SCh	NAS	
185	*Fusarium oxysporum*	46.39	58.23	20.24
22	Mortierellales (or)	12.20	0.94	37.51
414	*Preussia* (ge)	7.95	18.74	54.08
539	No Hit	8.21	0.12	66.48
538	Unclassified	7.30	0.53	76.87
245	Chaetomiaceae (fa)	4.35	7.00	84.45
65	*Trichocladium* (ge)	4.60	7.01	89.06
416	*Aspergillus versicolor*	1.10	0.04	90.69
353	*Chaetomium globosum*	0.41	1.00	91.99
162	*Penicillium lanosum*	0.78	0.13	93.00
382	*Alternaria chartarum*	1.16	1.32	93.94
174	*Mortierella alpina*	0.53	0.20	94.44
336	*Fusarium* (ge)	0.70	0.64	94.94
512	Unclassified	0.21	0.43	95.29
477	*Fusarium* (ge)	0.46	0.32	95.60
252	*Fusarium solani*	0.21	0.03	95.88
120	*Trichoderma atroviride*	0.42	0.36	96.10
402	*Preussia* (ge)	0.17	0.17	96.29
39	*Schizothecium inaequale*	0.16	0.21	96.47
429	*Preussia* (ge)	0.03	0.14	96.64

		SBSFL	NAS	
185	*Fusarium oxysporum*	63.11	58.23	33.79
414	*Preussia* (ge)	11.40	18.74	53.18
245	Chaetomiaceae (fa)	4.15	7.00	63.46
65	*Trichocladium* (ge)	4.63	7.01	68.95
120	*Trichoderma atroviride*	2.52	0.36	73.40
117	No Hit	1.71	0.21	76.48
353	*Chaetomium globosum*	1.95	1.00	79.44
472	Ascomycota (ph)	1.51	0.07	82.40
319	No Hit	0.88	0.00	84.20
382	*Alternaria chartarum*	0.98	1.32	85.68
515	Hypocreales (or)	0.65	0.04	86.93
22	Mortierellales (or)	0.42	0.94	88.05
336	*Fusarium* (ge)	0.36	0.64	88.79
78	*Trichoderma harzianum*	0.40	0.06	89.47
538	Unclassified	0.23	0.53	90.10
286	No Hit	0.29	0.00	90.70
530	No Hit	0.29	0.00	91.29
39	*Schizothecium inaequale*	0.45	0.21	91.78
294	*Acremonium alcalophilum*	0.22	0.00	92.23
512	Ascomycota (ph)	0.27	0.43	92.64

^†^ Cumulative contribution of this and all previous species in list;

ph, phylum; cl, class; or, Order; fa, family; ge, genus.

The fungal OTUs that were most enriched in BSFL frass amended soils (SBSFL) compared to unamended soils (NAS) were species of *Fusarium oxysporum* (OTU 185), *Chaetomium globosum* (353), *Schizothecium inaeguale* (OTU 39), *Acremonium alcalophilum* (OTU 294), *Trichioderma harizarum* (OUT 78) and *T. atroviride* (OTU 120), phylum *Ascomycota* (OTU 472) and order *Hypocreales* (OTU 515) (Table 4).

### 3.5 Characterization of the soil bacterial community

#### 3.5.1 Diversity and structure of the bacterial community

Pielou evenness index was significantly affected by the incubation, treatment and their interaction effect (*P < 0.05*, Table 3). This index value was highest for the non‐incubated soil amended with compost and lowest for the soils amended with chitin either incubated or non‐incubated. Non incubated soil amended with compost showed significantly higher evenness index than the incubated one. Shannon diversity index was affected by the treatment and the incubation of the soils (*P < 0.05*, Table 3). Similarly to the Evenness index, diversity had the highest value for the non‐incubated soils amended with compost and lowest value in the non‐incubated soils amended with chitin. Incubated soils did not differ significantly in Shannon index (Table 3).

**Figure 5:**
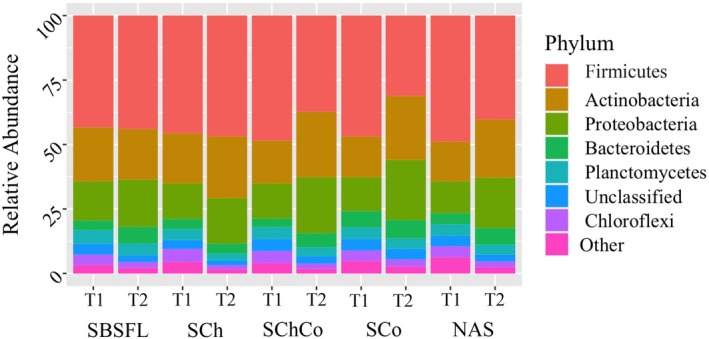
Relative abundance (%) of the main bacterial phylum measured in the incubated (T1) and non‐incubated soil (T2) non‐amended (NAS) or amended with frass from black soldier fly larvae (SBSFL), chitin (SCh), compost (SCo) or compost and chitin (SChCo).Figure 5. Relative abundance (%) of the main fungal classes measured in the incubated (T1) and non‐incubated soil (T2) non‐amended (NAS) or amended with frass from black soldier fly larvae (SBSFL), chitin (SCh), compost (SCo) or compost and chitin (SChCo).

The most abundant bacterial phyla were Firmicutes, Actinobacteria, Proteobacteria, Bacteroidetes, Planctomycetes, and Chloroflexi (Fig. 5, Table S2). The relative abundance of Firmicutes, and Proteobacteria phyla were significantly affected by the incubation period, amendment type and their interaction (*P < 0.05*). Those of Bacteroidetes and Planctomycetes, were significantly affected by the incubation and amendment type (*P < 0.05*). Finally, Actinobacteria and Chloroflexi were significantly affected by the incubation and the interaction between incubation and amendment type (*P < 0.05*). Firmicutes were lower and actinobacteria higher in the non‐incubated soil amended with compost with or without chitin, but it was recovered to natural levels in soils after the incubation (Fig. 5, Table S2). Proteobacteria and Chloroflexi relative abundances were significantly decreased and increased, respectively, after incubation in all amended soils (*P < 0.05*).

**Figure 6:**
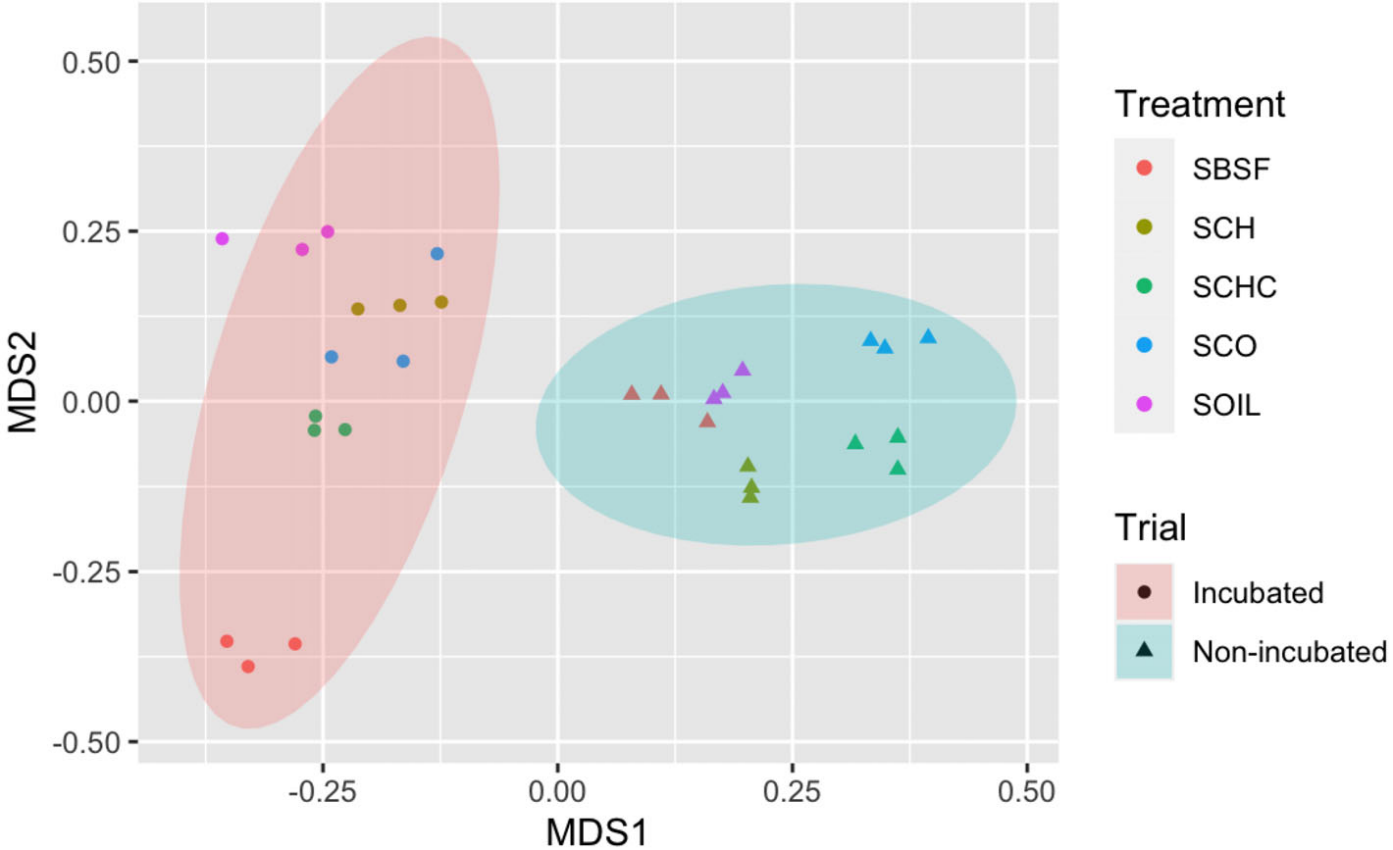
Non‐metric Multidimensional Scaling (NMDS) of the Bray Curtis dissimilarity of the Bacterial community in the non‐incubated and incubated soil, non‐amended (NAS) or amended with frass from black soldier fly larvae (SBSFL) frass, chitin (SCh), compost (SCo) or compost and chitin (SChC). Ellipses represent 95% confidence intervals for the centroids associated with each cluster related to the samples that were incubated 4 months (Trial 1) or non‐incubated (Trial 2).

Finally, the NMDS plot of the bacterial community showed significant differentiation within treatments and between the incubated and non‐incubated soils (Fig. 6). Soils incubated with BSFL frass showed the most differentiated microbial community structure compared to any other treatment.

#### 3.5.2 Similarity percentages (Simper) of the main OTUs of the bacterial community in the incubated non‐amended soil compared to the soils amended with BSFL frass

The OTUs that increased in relative abundance in SCh relative to NAS belonged to the genera *Arthrobacter*, *Thermomonas, Nocardioides, Sphaerobacter, Streptomyces* (OTUs 7132 and 4511), *Bacillus* (OTU 1205), *Nitrosospira, Sphingomonas, Chloroflexi* phylum and *Rubrobacterales* orden (OTUs 1467 and 1398) (Table 5).

**Table 5:** Similarity Percentages (Simper) of the 20 top bacteria OTUs of the incubated soil (trial 1) non‐amended compared to the soils amended with chitin (SCh) or black soldier fly larvae frass (SBSFL)

OTU	Genus	Relative abundance (%)		
		SCh	Soil	CumSum (%)
2465	*Bacillus*	22.46	24.03	5.23
7273	*Arthrobacter*	3.38	2.11	8.59
6240	*Bacillus*	7.60	8.83	11.88
6516	*Thermomonas*	1.44	0.91	13.28
7229	Planctomycetia (cl)	0.25	0.73	14.62
6429	*Nocardioides*	1.56	1.06	15.94
5127	*Sphaerobacter*	0.83	0.35	17.20
7132	*Streptomyces*	0.50	0.09	18.28
1205	*Bacillus*	4.32	4.20	19.32
5294	Chloroflexi (ph)	0.72	0.34	20.33
8519	*Nitrosospira*	0.69	0.34	21.26
3378	*Flavisolibacter*	1.11	1.30	22.18
1395	*Bacillus*	1.02	1.33	23.09
4511	*Streptomyces*	0.85	0.50	24.01
7824	No Hit	0.23	0.34	24.91
1467	Rubrobacterales (or)	0.76	0.47	25.79
1398	Rubrobacterales (or)	0.94	0.78	26.56
5449	*Planctomyces*	0.29	0.01	27.32
1864	*Flavisolibacter*	0.49	0.65	28.08
7863	*Sphingomonas*	0.35	0.12	28.71

		SBSFL	Soil	
2465	*Bacillus*	13.76	24.03	12.85
6425	*Streptomyces*	3.25	0.02	16.89
1205	*Bacillus*	5.45	4.20	18.45
775	Planctomycetia (cl)	0.91	0.00	19.58
5795	No Hit	0.88	0.01	20.67
5449	*Planctomyces*	0.81	0.01	21.68
7273	*Arthrobacter*	1.33	2.11	22.65
3214	*Mesorhizobium*	0.76	0.05	23.54
6240	*Bacillus*	9.08	8.83	24.37
1574	*Xanthomonas*	0.66	0.02	25.18
5675	*Bacillus*	0.64	0.00	25.98
7229	Planctomycetia (cl)	0.09	0.73	26.79
7547	*Sphingomonas*	0.84	0.22	27.55
6854	*Bacillus*	0.86	0.26	28.30
8264	Acidobacteriales (or)	0.18	0.76	29.02
7132	*Streptomyces*	0.62	0.09	29.69
1105	*Singulisphaera*	0.45	0.98	30.35
3524	Planctomycetia (cl)	0.72	0.21	30.99
6803	Rubrobacterales (or)	0.51	0.00	31.62
5230	*Moorella*	0.50	0.00	32.25

^†^ Cumulative contribution of this and all previous species in list. ph, phylum; cl, class; or, Order; fa, family.

As for the BSFL frass amended soil, the OTUs that increased their relative abundance with respect to the unamended soil (NAS) were *Streptomyces* (OTUs 6245, 7132), *Bacillus* (OTUs 1205, 6240, 5675, and 6854) *Planctomyces*, *Mesorhizobium*, *Xanthomonas*, *Sphingomonas*, Planctomycetia (OTUs 775 and 3524), order *Rubrobacterales*, and *Moorella*.

## 4 DISCUSSION

This study evaluated the impact of the addition of chitin‐rich amendments with long‐term incubation (4 months) and without incubation (only one week of incubation to allow *Fol* to establish in the soil) on the reduction of disease in lettuce caused by *Fol*. To elucidate the cause of disease suppression, the chemical and microbiological properties of the soil were studied. Special interest was paid to understanding the change in the microbial communities commonly known as biological control agents. The goal was to shed some light on the ability of BSFL frass suppress the target pathogen.

The results showed a high capacity of soils incubated for 4 months with chitin and BSFL frass to promote soil suppressiveness against *Fol*. In line with our results, the disease severity reduction achieved in soils amended with BSFL frass was also demonstrated against different pathogenic fungi such as *F. oxysporum* f. *sp. lycopersici* (Sacc.) Snyder y Hans, *Botrytis cinerea* Pers, *A. solani* Sorauer, *Sclerotinia sclerotiorum* (Lib.) de Bary and *Rhizoctonia solani* J. G. Kühn.^14^, ^15^, ^16^, ^30^

The use of BSFL as a soil improver has advantages and disadvantages. On the one hand, it can produce phytotoxic effects if the excrement has not been previously stabilized.^22^ However, one of the advantages of chitin‐rich soil improves is that, when applied at certain rates, it can increase lettuce fresh biomass by 124%.^19^ In our study, the addition of the amendments slightly affected the electrical conductivity and pH of the soils incubated in the short and long term, but this did not negatively affect the development of the lettuce nor positively affect the increase in its biomass (trial 3). Therefore, phytotoxic effects of the amendments were ruled out. All this seems to confirm that the effects observed on lettuce growth in trials 1 and 2 were due to the infection produced by *Fol* in the lettuce plants.

It is known that the addition of chitin amendments can modify soil microbial communities^11^, ^31^ promoting suppressive effects on plant diseases.^32^ In our study, we observed that the different amendments have a different impact on the microbial community and that this effect was more important after 4 months of incubation. Furthermore, the changes in the diversity index of the microbial community and in the structure of the bacterial community were more pronounced than in the fungal community, which highlights the resistance of the fungal microbial community. Many of these microorganisms have chitinolytic activity,^33^ that is, they potentially act by degrading the cell walls of fungi.^33^, ^34^ In addition, plants can recognize chitin oligosaccharides in the substrate and activate the expression of defense‐related genes^35^ to prevent colonization by pathogens. This includes the production of antimicrobial compounds and the strengthening of cell walls.^36^ These events could have been responsible for the reduction of *Fol* propagules and the severity of the disease in soils incubated with chitin and BSFL for 4 months. In contrast to these results, an increase in *Fol* propagules was observed in soils amended with chitin but not incubated. This increase in *F. oxysporum* propagules has been widely described by many authors.^37^, ^38^, ^39^ This can be attributed to the fact that *F. oxysporum* is a saprophytic organism that can feed on added organic matter.

### 4.1 Fungal biocontrol agents

Microorganisms involved in the control of microbial and fungal diseases are commonly known as biological control agents. The SIMPER analysis of the OTUs reported the common presence of some of these microorganisms in soils incubated with chitin and BSFL.

Among the results, different species of *Trichoderma spp*. were found, which are known for their ability to suppress the pathogenic activity of *Fusarium spp*. thanks to the synthesis of antibiotic compounds and lytic enzymes.^40^, ^41^

*Fusarium oxysporum* was also found in soils amended with chitin and BSFL. This was expected because pathogenic and non‐pathogenic species of *F. oxysporum* can coexist in the soil.^42^ In addition, non‐pathogenic species of *F. oxysporum* may have antagonistic activity against Fusarium wilt. This is because saprophytic *F. oxysporum* colonizes the roots of plants, occupying the biological niche of the pathogenic forms, activates the mechanisms of iron and nutrient mobility, and induces the defense systems of the plants.^43^ Gilardi observed a significant reduction in the severity of the disease and an increase in the fresh weight of lettuce compared to the inoculated and untreated control when treated with non‐pathogenic strains of *F. oxysporum*, so the presence of this fungus in soils amended with chitin and BSFL could be promoting the control of Fusarium wilt.^44^

The genera *Aspergillus* and *Mortierella* were enriched in chitin‐incubated soils. Both fungi are involved in the degradation of cell wall chitin^44^, ^45^ and are associated with suppressive soils.^46^ *Morteriella* had also previously been enriched in chitin‐modified soil^47^ and its presence has been linked to enhanced disease suppressiveness following soil fumigation and biofertilizer application.^48^ These fungi are known to be active against species of the genus *Fusarium*.^49^, ^50^ In addition, *Mortierella spp*. has a positive influence on soil activities and plant growth parameters.^51^

*Chaetomium globosum* was enriched in soils incubated with BSFL. These microorganisms produce antifungal compounds such as benzothiazole. The compounds produced by this species are known to act against species of the genus *Fusarium in vitro*.^52^

### 4.2 Bacterial biocontrol agents

In our SIMPER analyses, numerous bacteria that could be acting as biocontrol agents were found to be enriched in soil incubated with chitin (SCh) and black soldier fly larvae (BSFL) compared to unmodified soil.

In soils incubated with chitin and BSFL, an increase in the relative abundance of the genera *Bacillus spp*. and *Streptomyces spp*. was observed. These are known as BCAs and are commonly found in disease‐suppressing soils. Some *Bacillus* species are associated with plant roots and are beneficial to their growth.^53^, ^54^ As for bacteria of the genus *Streptomyces*, their method of action is based on the production of secondary metabolites, by competition for resources such as carbon and iron,^55^, ^56^ and by induction of the plant's defense system.^57^, ^58^ These microorganisms are very effective against pathogenic forms of *F. oxysporum*.^55^, ^59^, ^60^

In soils incubated with chitin, bacteria of the genera *Arthrobacter*, *Sphingomonas* and *Chloroflexi* were found. These microorganisms have chitinase activity or participate in the activation of the immune response of plants through jasmonic acid, salicylic acid, abscisic acid and ethylene.^60^, ^61^ In addition, the activity of bacteria of the genera *Arthrobacter* and *Chlorolexi* against Fusarium is well known.^59^ Therefore, any group of these bacteria could be involved in the suppressiveness of the soil against Fusarium wilt.

## 5 CONCLUSION

This study showed that incubation of chitin and BSFL frass in the soil has the capacity to promote suppressive soil against *Fol*, and, therefore, to reduce the severity and the population density of this pathogen. Chitin or BSFL frass concentration, incubation period or amendment rate are some of the parameters that could be tuned to improve this soil suppressiveness and should be investigated. The changes observed in the microbial soil community were more evident in the bacterial community than in the fungal community. Soils incubated with chitin and BSFL for 4 months showed a strong enrichment of fungal and bacterial OTUs recognized as biological control agents. Therefore, the microbiota generated after the four‐month incubation period with these amendments could be involved in the suppressiveness acquired by the soil against *Fol*. This study contributes to the knowledge of the impact of chitin enriched amendments leading to new studies that could be more targeted on selected microorganisms to elucidate their role in soil suppression.

## CONFLICT OF INTEREST

The authors declare that they have no known competing financial interests or personal relationships that could have appeared to influence the work reported in this paper.

## AUTHOR CONTRIBUTIONS

Paloma Hernández‐Muñiz: Writing – review & editing, Writing – original draft, Visualization, Validation, Methodology, Investigation, Formal analysis, Data curation, Conceptualization. Celia Borrero: review & editing, Visualization, Project administration, Methodology, Investigation, Funding acquisition, Conceptualization. Manuel Avilés: review, Investigation, Formal analysis, Data curation, Conceptualization. Jesús D. Fernández‐Bayo: Writing – review & editing, Writing – original draft, Visualization, Validation, Methodology, Resources, Investigation, Formal analysis, Data curation, Resources, Funding acquisition, Conceptualization.

## Supporting information


**Table S1.** Relative abundance (%) of the main fungal classes measured in the non‐incubated (Non‐Inc) and incubated soil (Inc) non‐amended (SOIL) or amended with frass from black soldier fly larvae (SBSF), chitin (SCH), compost (SCO) or compost and chitin (SCHC).
**Table S2.** Relative abundance (%) of the main bacterial phyla measured in the non‐incubated (Non‐Inc) and incubated soil (Inc) non‐amended (SOIL) or amended with frass from black soldier fly larvae (SBSF), chitin (SCH), compost (SCO) or compost and chitin (SCHC).

## Data Availability

The data that support the findings of this study are available from the corresponding author upon reasonable request.

## References

[1] Matuo T and Motohashi S , On *Fusarium oxysporum* f.sp. *lactucae* n.f. causing root rot of lettuce. Trans, mycol Soc Japan 8:13–15 (1967).

[2] Gordon TR and Koike ST , Management of Fusarium wilt of lettuce. Crop Prot 73:45–49 (2015).

[3] Lloyd M and Gordon T , Growing for the future: collective action, land stewardship and soilborne pathogens in California strawberry production. Calif Agric 70:101–103 (2016).

[4] Avilés, M , Borrero, C and Trillas, MI . Review on compost as an inducer of disease suppression in plants grown in soilless culture. In: Sánchez Ferrer, A. (Eds.). Dynamic Soil, Dynamic Plant, Compost III. Global Science Books 5, special issue 2, Japan, pp:1–11 (2011).

[5] Baker KF and Cook RJ , Biological Control of Plant Pathogens. *American Phytopathological Society* , San Francisco, p. 433 (1974).

[6] Termorshuizen AJ and Jeger MJ , Strategies of soilborne plant pathogenic fungi in relation to disease suppression. Fungal Ecol 1:108–114 (2008).

[7] Borrero C , Trillas MI , Ordovás J , Tello JC and Avilés M , Predictive factors for the suppression of Fusarium wilt of tomato in plant growth media. Phytopathology 94:1094–1101 (2004). 18943798 10.1094/PHYTO.2004.94.10.1094

[8] Schlatter D , Kinke L , Thomashow L , Weller D and Paulitz T , Disease suppressive soils: new insights from the soil microbiome. Phytopathology 107:1284–1297 (2017). 28650266 10.1094/PHYTO-03-17-0111-RVW

[9] Gooday GW , The ecology of chitin degradation, in Advances in Microbial Ecology. Springer US, Boston, MA, pp. 387–430 (1990).

[10] Pusztahelyi T , Chitin and chitin‐related compounds in plant–fungal interactions. Mycology 9:189–201 (2018). 30181925 10.1080/21501203.2018.1473299 PMC6115883

[11] Cretoiu MS , Korthals GW , Visser JH and van Elsas JD , Chitin amendment increases soil suppressiveness toward plant pathogens and modulates the actinobacterial and oxalobacteraceal communities in an experimental agricultural field. Appl Environ Microbiol 79:5291–5301 (2013). 23811512 10.1128/AEM.01361-13 PMC3753968

[12] Oka Y , Mechanisms of nematode suppression by organic soil amendments ‐a review. Appl Soil Ecol 44:101–115 (2010).

[13] Surendra KC , Tomberlin JK , van Huis A , Cammack JA , Heckmann L and Khanal SK , Rethinking organic wastes bioconversion: evaluating the potential of the black soldier fly (*Hermetia illucens* (L.)) (Diptera: Stratiomyidae) (BSF). Waste Manag 117:58–80 (2020). 32805602 10.1016/j.wasman.2020.07.050

[14] Arabzadeh G , Delisle‐Houde M , Tweddell RJ , Deschamps MH , Dorais M , Lebeuf Y *et al*., Diet composition influences growth performance, bioconversion of black soldier fly larvae: agronomic value and in vitro biofungicidal activity of derived frass. Agronomy 12:1765 (2022).

[15] Arabzadeh G , Delisle‐Houde M , Vandenberg GW , Deschamps MH , Dorais M , Derome N *et al*., Suppressive effect of black soldier fly larvae frass on Fusarium wilt disease in tomato plants. Insects 15:613 (2024). 39194818 10.3390/insects15080613 PMC11354714

[16] Arabzadeh G , Antifungal properties of black soldier fly larval frass: impact on plant pathogens control. PhD thesis, Quebec, Canada (2024).

[17] Kemboi VJ , Kipkoech C , Njire M , Were S , Lagat MK , Ndwiga F *et al*., Biocontrol potential of chitin and chitosan extracted from black soldier fly pupal exuviae against bacterial wilt of tomato. Microorganisms 10:165 (2022). 35056613 10.3390/microorganisms10010165 PMC8780822

[18] Wantulla M , van Loon JJ and Dicke M , Soil amendment with insect exuviae causes species‐specific changes in the rhizosphere bacterial community of cabbage plants. Appl Soil Ecol 188:104854 (2023).

[19] Debode J , De Tender C , Soltaninejad S , Van Malderghem C , Haegeman A , Van der Linden I *et al*., Chitin mixed in potting soil alters lettuce growth, the survival of zoonotic bacteria on the leaves and associated rhizosphere microbiology. Front Microbiol 7:565 (2016). 27148242 10.3389/fmicb.2016.00565 PMC4838818

[20] van de Zande EM , Wantulla M , van Loon JJ and Dicke M , Soil amendment with insect frass and exuviae affects rhizosphere bacterial community, shoot growth and carbon/nitrogen ratio of a brassicaceous plant. Plant and Soil 495:631–648 (2024).

[21] Inderbitzin P , Ward J , Barbella A , Solares N , Izyumin D , Burman P *et al*., Soil microbiomes associated with Verticillium wilt‐suppressive broccoli and chitin amendments are enriched with potential biocontrol agents. Phytopathology 108:31–43 (2018). 28876209 10.1094/PHYTO-07-17-0242-R

[22] Axelrod R , Palma Miner L , VanderGheynst JS , Simmons CW and Fernandez‐Bayo JD , Soil application of almond residue biomass following black soldier Fly larvae cultivation. Front Sustain Food Syst 5:664635 (2021).

[23] Fang X , You MP and Barbetti MJ , Reduced severity and impact of Fusarium wilt on strawberry by manipulation of soil pH, soil organic amendments and crop rotation. Eur J Plant Pathol 134:619–629 (2012).

[24] Campbell CL and Madden LV , Introduction to Plant Disease Epidemiology. Wiley, New York, p. 532 (1990).

[25] Pastrana AM , Shea EA , Fernandez‐Bayo JD , Allison B , Watson DC , Toniato J *et al*., Impact of biosolarization with almond hull and shell amendments for the control of *Fusarium oxysporum* f. sp. *lactucae* in a lettuce/tomato cropping system. Crop Prot 156:105925 (2022).

[26] Komada H , Development of a selective medium for quantitative isolation of *Fusarium oxysporum* from natural soil. Rev Plant Prot Res 8:114–124 (1975).

[27] Scott JC , Kirkpatrick SC and Gordon TR , Variation in susceptibility of lettuce cultivars to Fusarium wilt caused by *Fusarium oxysporum* f.sp *lactucae* . Plant Pathol 59:139–146 (2010).

[28] Oksanen J , Kindt R , Legendre P , O’Hara B , Henry M and Stevens H , The vegan package. Community Ecology Package 10:719 (2007) http://cran.r-project.org/, http://vegan.r-forge.r-project.org/.

[29] Clarke KR , Non‐parametric multivariate analyses of changes in community structure. Aust J Ecol 18:117–143 (1993).

[30] Arabzadeh G , Delisle‐Houde M , Vandenberg GW , Derome N , Deschamps MH , Dorais M *et al*., Assessment of antifungal/anti‐oomycete activity of frass derived from black soldier fly larvae to control plant pathogens in horticulture: involvement of *Bacillus velezensis* . Sustainability 15:10957 (2023).

[31] Iwasaki Y , Ichino T and Saito A , Transition of the bacterial community and culturable chitinolytic bacteria in chitin‐treated upland soil: from Streptomyces to methionine‐auxotrophic Lysobacter and other genera. Microbes environ 35:ME19070 (2020). 31932540 10.1264/jsme2.ME19070 PMC7104288

[32] Ootsuka E , Iwasaki Y , Takagi K and Saito A , LMC60, a material containing low‐molecular‐weight chitin: degradation and effects on soil microorganisms in incubated upland soil. Soil Sci Plant Nutr 67:389–399 (2021).

[33] Bell AA , Hubbard JC , Liu L , Davis RM and Subbarao KV , Effects of chitin and chitosan on the incidence and severity of Fusarium yellows of celery. Plant Dis 82:322–328 (1998). 30856866 10.1094/PDIS.1998.82.3.322

[34] Sharp RG , A review of the applications of chitin and its derivatives in agriculture to modify plant‐microbial interactions and improve crop yields. Agronomy 3:757–793 (2013).

[35] Bowman SM and Free SJ , The structure and synthesis of the fungal cell wall. Bioessays 28:799–808 (2006). 16927300 10.1002/bies.20441

[36] Riseh RS , Vazvani MG , Vatankhah M and Kennedy JF , Chitin‐induced disease resistance in plants: a review. Int J Biol Macromol 266:131105 (2024). 38531527 10.1016/j.ijbiomac.2024.131105

[37] Butler DM , Rosskopf EN , Kokalis‐Burelle N , Albano JP , Muramoto J and Shennan C , Exploring warm‐season cover crops as carbon sources for anaerobic soil disinfestation (ASD). Plant and Soil 355:149–165 (2012).

[38] Hernández‐Muñiz P , Borrero C , Ordóñez‐Martín J , Pastrana AM and Avilés M , Optimization of the use of industrial wastes in anaerobic soil disinfestation for the control of Fusarium wilt in strawberry. Plan Theory 12:3185 (2023). 10.3390/plants12183185 PMC10534816 37765349

[39] Mazzola M , Muramoto J and Shennan C , Anaerobic disinfestation induced changes to the soil microbiome, disease incidence and strawberry fruit yields in California field trials. Appl Soil Ecol 127:74–86 (2018).

[40] Gezgin Y , Maral Gül D , Sözer Şenşatar S , Kara C , Sargın S , Sukan F *et al*., Evaluation of *Trichoderma atroviride* and *Trichoderma citrinoviride* growth profiles and their potentials as biocontrol agent and biofertilizer. Turk J Biochem 45:163–175 (2020).

[41] Özkale E , Yörük E , Budak M and Korkmaz EM , *Trichoderma atroviride* suppresses *Fusarium graminearum* by altering primary and secondary metabolite biosynthesis profiling. Plant Pathol 72:1428–1441 (2023).

[42] Zhou X and Wu F , Dynamics of the diversity of fungal and Fusarium communities during continuous cropping of cucumber in the greenhouse. FEMS Microbiol Ecol 80:469–478 (2012). 22273443 10.1111/j.1574-6941.2012.01312.x

[43] Hoitink HA and Locke JC , CHAPTER 11: an integrated approach to biological control of Fusarium species in containerized crops, in Fusarium Wilts of Greenhouse Vegetable and Ornamental Crops. The American Phytopathological Society, St. Paul, MN, pp. 109–115 (2012).

[44] Gilardi G , Vasileiadou A , Garibaldi A and Gullino ML , The effects of biological control agents, potassium phosphite and calcium oxide on race 1 of *Fusarium oxysporum* f. sp. *lactucae* of lettuce in closed soilless cultivation systems. J Phytopathol 170:626–634 (2022).

[45] Dong M , Wang S , Xiao G , Xu F , Hu W , Li Q *et al*., Cellulase production by aspergillus fumigatus MS13. 1 mutant generated by heavy ion mutagenesis and its efficient saccharification of pretreated sweet sorghum straw. Process Biochem 84:22–29 (2019).

[46] Swiontek Brzezinska M , Jankiewicz U , Burkowska A and Walczak M , Chitinolytic microorganisms and their possible application in environmental protection. Curr Microbiol 68:71–81 (2014). 23989799 10.1007/s00284-013-0440-4 PMC3889922

[47] Siegel‐Hertz K , Edel‐Hermann V , Chapelle E , Terrat S , Raaijmakers JM and Steinberg C , Comparative microbiome analysis of a Fusarium wilt suppressive soil and a Fusarium wilt conducive soil from the Châteaurenard region. Front Microbiol 9:568 (2018). 29670584 10.3389/fmicb.2018.00568 PMC5893819

[48] Randall TE , Fernandez‐Bayo JD , Harrold DR , Achmon Y , Hestmark KV , Gordon TR *et al*., Changes of *Fusarium oxysporum* f. sp. *lactucae* levels and soil microbial community during soil biosolarization using chitin as soil amendment. PLoS One 15:e0232662 (2020). 32369503 10.1371/journal.pone.0232662 PMC7199936

[49] Israel S and Lodha S , Biological control of *Fusarium oxysporum* f. sp. *cumini* with *Aspergillus versicolor* . Phytopathol Mediterr 44:3–11 (2005).

[50] Shen Z , Xue C , Taylor PW , Ou Y , Wang B , Zhao Y *et al*., Soil pre‐fumigation could effectively improve the disease suppressiveness of biofertilizer to banana Fusarium wilt disease by reshaping the soil microbiome. Biol Fertil Soils 54:793–806 (2018).

[51] Ozimek E and Hanaka A , Mortierella species as the plant growth‐promoting fungi present in the agricultural soils. Agri 11:7 (2021).

[52] Sangeetha C , Kiran Kumar N , Krishnamoorthy AS and Harish S , Biomolecules from *Chaetomium globosum* possessing antimicrobial compounds potentially inhibits Fusarium wilt of tomato. Appl Biochem Biotechnol 196:2196–2218 (2024). 37490243 10.1007/s12010-023-04620-9

[53] Idriss EE , Makarewicz O , Farouk A , Rosner K , Greiner R , Bochow H *et al*., Extracellular phytase activity of *Bacillus amyloliquefaciens* FZB45 contributes to its plant‐growth‐promoting effect. Microbiol 148:2097–2109 (2002). 10.1099/00221287-148-7-2097 12101298

[54] Cha JY , Han S , Hong HJ , Cho H , Kim D , Kwon Y *et al*., Microbial and biochemical basis of a Fusarium wilt‐suppressive soil. ISME J 10:119–129 (2016). 26057845 10.1038/ismej.2015.95 PMC4681868

[55] Kinkel LL , Schlatter DC , Bakker MG and Arenz BE , *Streptomyces* competition and co‐evolution in relation to plant disease suppression. Res Microbiol 163:490–499 (2012). 22922402 10.1016/j.resmic.2012.07.005

[56] Conn VM , Walker AR and Franco CMM , Endophytic actinobacteria induce defense pathways in *Arabidopsis thaliana* . Mol Plant Microbe Interact 21:208–218 (2008). 18184065 10.1094/MPMI-21-2-0208

[57] Kurth F , Mailänder S , Bönn M , Feldhahn L , Herrmann S and Große I , *Streptomyces*‐induced resistance against oak powdery mildew involves host plant responses in defense, photosynthesis, and secondary metabolism pathways. Mol Plant Microbe Interact 27:891–900 (2014). 24779643 10.1094/MPMI-10-13-0296-R

[58] El‐Fallal AA , Abou‐Dobara MI , El‐Sayed AK , Shaban E and Mousa MM , Antifungal activity of two streptomyces species isolated from egyptian soil against some phytopathogenic fungi. J Microbiol Biotechnol Food Sci 14:e10665 (2024).

[59] Zhao M , Yuan J , Zhang R , Dong M , Deng X , Zhu C *et al*., Microflora that harbor the NRPS gene are responsible for Fusarium wilt disease‐suppressive soil. Appl Soil Ecol 132:83–90 (2018).

[60] Morrissey RF , Dugan EP and Koths JS , Chitinase production by an *Arthrobacter* sp. *lysing* cells of *Fusarium roseum* . Soil Biol Biochem 8:23–28 (1976).

[61] Yang F , Jiang H , Chang G , Liang S , Ma K , Cai Y *et al*., Effects of rhizosphere microbial communities on cucumber Fusarium wilt disease suppression. Microorganisms 11:1576 (2023). 37375078 10.3390/microorganisms11061576 PMC10301182

