# Supplementary material for: Perspectives on microbial community changes produced by Hermitia illucens frass and their impact on soil suppression against Fusarium oxysporum f. sp. lactucae
Source: Pest Manag Sci. 2025 Jul 8;81(10):6832–42. doi: 10.1002/ps.70036 (PMC12441770; doi:10.1002/ps.70036)
Supplement: Supplementary file 1 — Table S1. Relative abundance (%) of the main fungal classes measured in the non‐incubated (Non‐Inc) and incubated soil (Inc) non‐amended (SOIL) or amended with frass from black soldier fly larvae (SBSF), chitin (SCH), compost (SCO) or compost and chitin (SCHC). Table S2. Relative abundance (%) of the main bacterial phyla measured in the non‐incubated (Non‐Inc) and incubated soil (Inc) non‐amended (SOIL) or amended with frass from black soldier fly larvae (SBSF), chitin (SCH), compost (SCO) or compost and chitin (SCHC). [file PS-81-6832-s001.docx]

**Perspectives on microbial community changes in chitin-exposed amendments and their impact on soil suppression against *Fusarium oxysporum* f. sp. *lactucae***

Paloma Hernández-Muñiz1, Celia Borrero1, Manuel Avilés1, Jesús D. Fernández-Bayo2,3

^1^Departamento de Agronomía, Escuela Técnica Superior de Ingeniería Agronómica de Sevilla, Universidad de Sevilla, Sevilla, Spain.

^2^Department of Biological and Agricultural Engineering. University of California Davis, California, USA.

^3^Department of Soil Science and Agricultural Chemistry. University of Granada, Granada, Spain.

**Table S1. Relative abundance (%) of the main fungal classes measured in the non-incubated (Non-Inc) and incubated soil (Inc) non-amended (SOIL) or amended with frass from black soldier fly larvae (SBSF), chitin (SCH), compost (SCO) or compost and chitin (SCHC).**

|  | **Sordariomycetes** | **Dothideomycetes** | **Mortierellomycetes** | **Eurotiomycetes** | **Leotiomycetes** | **Tremellomycetes** |
| --- | --- | --- | --- | --- | --- | --- |
| SOIL_Inc | 75.86±9.61 | 20.45±8.89 | 1.21±0.56b | 0.26±0.09b | 0.12±0.03 | 0.05±0.03cd* |
| SOIL_No_Inc | 78.11±5.08 | 15.43±3.33 | 2.24±0.66b | 0.51±0.23b | 0.21±0.08 | 0.25±0.1ab |
| SCO_Inc | 77.92±0.65 | 13.1±2.44 | 2.04±0.22b | 0.31±0.07b | 0.18±0.02 | 0.06±0.03cd* |
| SCO_No_Inc | 75.56±11.48 | 16.77±80 | 1.76±0.16b | 0.31±0.12b | 0.19±0.07 | 0.32±0.03a |
| SCH_Inc | 58.59±13.55* | 9.32±3.09 | 13.07±4.9a* | 2.09±0.86b* | 0.09±0.03* | 0.06±0.01cd* |
| SCH_No_Inc | 81.75±4.50 | 11.82±2.96 | 2.01±0.67b | 0.46±0.11b | 0.18±0.01 | 0.18±0.06bc |
| SCHC_Inc | 62.89±9.50 | 13.23±4.01 | 4.67±1.15b* | 7.28±1.82a* | 0.22±0.03 | 0.06±0.02cd* |
| SCHC_No_Inc | 75.27±3.56 | 16.08±3.47 | 2.47±0.39b | 0.44±0.15b | 0.19±0.07 | 0.21±0.02ab |
| SBSF_Inc | 80.24±12.35 | 12.69±8.14 | 0.46±0.23b | 0.19±0.1b | 0.28±0.17 | 0.04±0.02d* |
| SBSF_No_Inc | 75.87±12.56 | 14.94±8.12 | 2.73±1.44b | 0.6±0.52b | 0.22±0.14 | 0.18±0.06bc |

Different letters indicate significant differences among treatments within the same phyla (P<0.05) using Tukey High Significant Difference test. * Indicates significant differences between incubated and non-incubated samples of the same treatment and phylum using t-student test.

**Table S2. Relative abundance (%) of the main bacterial phyla measured in the non-incubated (Non-Inc) and incubated soil (Inc) non-amended (SOIL) or amended with frass from black soldier fly larvae (SBSF), chitin (SCH), compost (SCO) or compost and chitin (SCHC).**

|  | **Firmicutes** | **Actinobacteria** | **Proteobacteria** | **Bacteroidetes** | **Planctomycetes** | **Chloroflexi** | **Acidobacteria** |
| --- | --- | --- | --- | --- | --- | --- | --- |
| SOIL_Inc | 48.97±4.67a* | 15.33±1.33g* | 12.52±1.22f* | 4.1±0.74bcd* | 4.62±1.03ab | 4.22±0.26a* | 2.96±0.4a* |
| SOIL_No_Inc | 40.36±3.69abc | 22.51±2.04abcd | 19.46±1.31bc | 6.64±0.17ab | 3.83±0.18abc | 2.3±0.1b | 1.25±0.09cd |
| SCO_Inc | 46.8±6.1a* | 15.81±2.09fg* | 13.35±1.79f* | 6.31±2.46abc | 4.37±1.2ab | 4.03±0.71a* | 2.42±0.16b* |
| SCO_No_Inc | 31.13±0.63c | 24.8±1.91ab | 23.39±1.22a | 7.12±0.58a | 4.14±0.12abc | 2.72±0.22b | 0.95±0.11cde |
| SCH_Inc | 45.67±1.5ab | 19.38±0.88defg* | 13.99±0.11ef* | 3.72±0.61cd | 4.27±0.66abc* | 5.07±0.78a* | 2.08±0.17b* |
| SCH_No_Inc | 46.9±1.31a | 23.85±0.8abc | 17.73±1.01cde | 3.78±0.29cd | 2.64±0.1c | 1.75±0.07b | 0.76±0.05de |
| SCHC_Inc | 48.41±2.31a* | 16.73±0.25efg* | 13.67±0.39f* | 3.2±0.71d* | 4.6±0.14ab* | 4.69±0.13a* | 1.41±0.19c* |
| SCHC_No_Inc | 37.25±3.5bc | 25.49±0.46a | 21.7±2.6ab | 5.42±0.41abcd | 3.45±0.49bc | 2.13±0.21b | 0.71±0.1e |
| SBSF_Inc | 43.40±1.59ab | 20.98±1.04bcde | 15.22±1.07def* | 3.56±0.43d | 5.27±0.13a* | 3.99±0.36a* | 0.74±0.08e* |
| SBSF_No_Inc | 43.95±2.99ab | 19.87±2.33cdef | 18.23±0.5bcd | 6.49±0.47ab | 4.5±0.44ab | 2.09±0.3b | 1.11±0.09cde |

Different letters indicate significant differences among treatments within the same phyla (P<0.05) using Tukey HSD test. * Indicates significant differences between incubated and non-incubated samples of the same treatment and phylum using t-student test.
